# The Interaction of the *Atm* Genotype with Inflammation and Oxidative Stress

**DOI:** 10.1371/journal.pone.0085863

**Published:** 2014-01-20

**Authors:** Yan Yang, Chin Wai Hui, Jiali Li, Karl Herrup

**Affiliations:** 1 Department of Neurology & Neurosciences, SOM E720, Case Western Reserve University, School of Medicine, Cleveland, Ohio, United States of America; 2 Division of Life Science, Hong Kong University of Science and Technology, Clear Water Bay, Kowloon, Hong Kong; 3 Department of Cell Biology and Neuroscience, Rutgers University, Piscataway, New Jersey, United States of America; Massachusetts General Hospital and Harvard Medical School, United States of America

## Abstract

In ataxia-telangiectasia (A–T) the death of neurons is associated with the loss of neuronal cell cycle control. In most *Atm^−/−^* mouse models, however, these cell cycle anomalies are present but the phenotype of neuronal cell loss found in humans is not. Mouse *Atm* ^−/−^ neurons re-enter a cell cycle and replicate their DNA, but they do not die – even months after initiating the cycle. In the current study, we explore whether systemic inflammation or hypoxia-induced oxidative stress can serve as second stressors that can promote cell death in ATM-deficient neurons. We find that after either immune or hypoxic challenge, the levels of cell cycle proteins – PCNA, cyclin A and cyclin B – are significantly elevated in cerebellar Purkinje cells. Both the number of cells that express cell cycle proteins as well as the intensity of the expression levels in each cell is increased in the stressed animals. The cell cycle-positive neurons also increasingly express cell death markers such as activated caspase-3, **γ**-H2AX and TUNEL staining. Interestingly, nuclear HDAC4 localization is also enhanced in *Atm* ^−/−^ Purkinje neurons after the immune challenge suggesting that both genetic and epigenetic changes in *Atm^−/−^* mice respond to environmental challenges. Our findings support the hypothesis that multiple insults are needed to drive even genetically vulnerable neurons to die a cell cycle-related cell death and point to either inflammation or oxidative stressors as potential contributors to the A−T disease process.

## Introduction

Ataxia-telangiectasia (A–T) is an autosomal recessive disorder characterized by immune deficiency, radiation sensitivity, sterility and enhanced risk of cancer. Affected individuals also develop a progressive neurodegeneration that strikes particularly hard at the Purkinje and granule cells of the cerebellar cortex. In advanced stages of the disease, neuronal cell loss continues and there is a substantial loss of motor function [1], [2]. The gene whose mutation leads to A–T encodes a PI3-kinase family member known as ATM (ataxia-telangiectasia mutated). As ATM is a key player in the DNA double strand break response, A−T symptoms such as cancer, immune dysfunction, radiosensitivity and sterility seem to follow logically. There are other phenotypes that are part of the full A−T syndrome, however. They include neuronal vesicle trafficking problems and LTP deficits [3], insulin signaling problems [4], [5], as well as defects in the histone epigenetic code [6], mitochondrial integrity [7] and the pentose phosphate pathway [8]. Structurally, ATM-deficient neurons are less able to develop a full dendritic structure in culture [9]. DNA damage repair defects could contribute to each of these problems. For example, ATM-deficient neurons are more sensitive than wild type to DNA damage induced by oxidation or genotoxic compounds such as etoposide, methotrexate and homocysteine [10] but the view is emerging that the neurological symptoms of A−T are a composite of dysfunctions in many different systems [11].

Our laboratory focuses on cell cycle control in the adult neuron and the relationship between an abortive cell cycle re-entry and neuronal cell death. We have shown, in both human A−T and two different mouse models of ATM deficiency that the neuronal re-expression of cell cycle proteins is associated with the death of Purkinje cells and striatal neurons [3], [6], [12], [13]. However, while A−T neurons express cell cycle proteins and replicate their DNA, afterwards they can and do survive for extended periods of time without undergoing cell death [12]. In the field of Alzheimer’s research, a similar observation has led to the speculation that the death of a cycling neuron requires a “two hit” process [14], [15]. In this study, we explore the possibility that environmental stresses such as an oxidative challenge or activation of the immune system might play such a role in the events of A–T neuronal cell death.

The physical status and activity of the ATM protein are known to be sensitive to oxidation [16], [17], and ATM deficient neurons are more sensitive to oxidative damage [9]. Although there is no reported evidence for an inflammatory process found in the brains of A–T individuals, the activation of both the peripheral and CNS immune systems are well known to have a profound influence on behavior and neuronal viability [18]. A peripheral immune challenge and the resulting cytokine ‘storm’ can alter the function of the brain to the point where delirium sets in. Further, in other neurodegenerative diseases such as Alzheimer’s chronic inflammation is both present and proposed to play a direct role in disease progression [19]–[22]. In the current work, we show that both of these environmental factors have important relevance for the symptoms of A–T. We show that cell cycle proteins in *Atm^−/−^* Purkinje cells are increased in mice exposed to either acute or chronic LPS injection. Sustained LPS treatment drives Purkinje and granule cells to become positive for cell death markers such as TUNEL, **γ**-H2AX and activated caspase-3. This correlation between inflammation, defective cell cycle regulation, and the initiation of neuronal death offers fresh insight into the question of why neurons die during the course of A–T.

## Results

### Cell Cycle Proteins Increase in Purkinje Cells of *Atm* ^−/−^ Mice Exposed to Secondary Stressors

Our previous work has shown that cell cycle proteins are elevated in Purkinje cells in both human A–T disease and the ATM-deficient mouse [12]. To determine why cycling mouse *Atm^−/−^* Purkinje cells do not die, we tested the hypothesis that a cell cycle-positive neuron, although weakened, might require a second stress to initiate the final death progress. Thus, we explored the roles of two possible stressors – hypoxia induced oxidative stress and LPS induced inflammation – as triggers that might induce cell death in the ‘cycling’ neurons.

**Figure 1:**
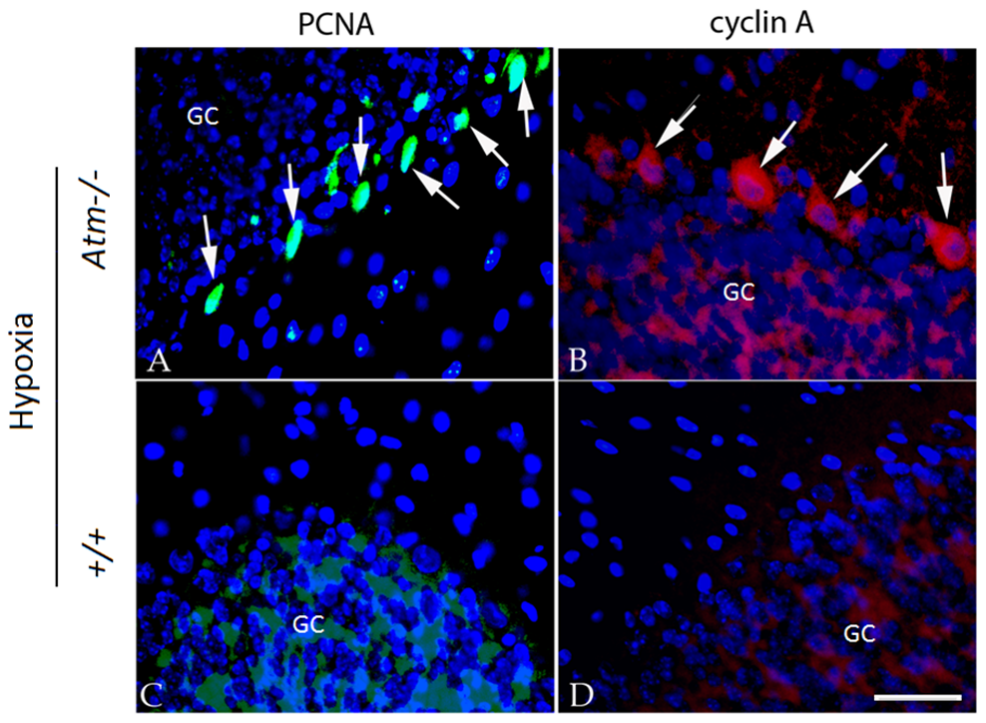
Cell cycle proteins increased in Purkinje cells of *Atm^−/−^* mice after hypoxia treatment. The representative pictures from each group (n = 3–4, repeated 3 times) were shown. Expression of PCNA (A, C) and cyclin A (B, D) increased after hypoxia treatment. In each panel, white arrows indicate the Purkinje cells that stained with cell cycle markers. GC = granule cell layer, Scale bar = 25 µm.

Mutant animals and wild-type controls were exposed 3 times to an atmosphere with reduced oxygen tension (8% O_2_) for 30 minutes with a 20-minute recovery period between exposures. Expression of PCNA (proliferating cell nuclear antigen – a component of the DNA replication complex – Figure 1A) and cyclin A (an S-phase cyclin – Figure 1B) were both increased in the nuclei of treated *Atm^−/−^* Purkinje cells, but not in wild type Purkinje cells (*Atm* ^+/+^) exposed to the same regimen (Figure 1C, D). Neurons in other brain regions that are cell cycle-negative in the untreated *Atm^−/−^* mouse were not noticeably affected by the low oxygen treatment. These findings suggest that the cell cycle status of the neurons in the *Atm^−/−^* brain renders them sensitive to additional environmental challenges.

### Chronic LPS-induced Inflammation Drives ATM-deficient Purkinje Cells towards Degeneration

To further test the validity of this idea, we compared three different types of LPS administration. By injecting LPS once a week for 4 weeks, we subjected the neurons to a recurrent inflammatory environment. To test for the effects of an acute inflammatory episode, we injected LPS once (0.5 mg/kg) but then waited 7 days before sacrificing the animals. Finally, to test for the immediate effects of a surge of inflammatory cytokines, we adopted a paradigm of daily administration of LPS (1 mg/kg) for 3–4 days followed by sacrifice and analysis after 24 hours.

**Figure 2:**
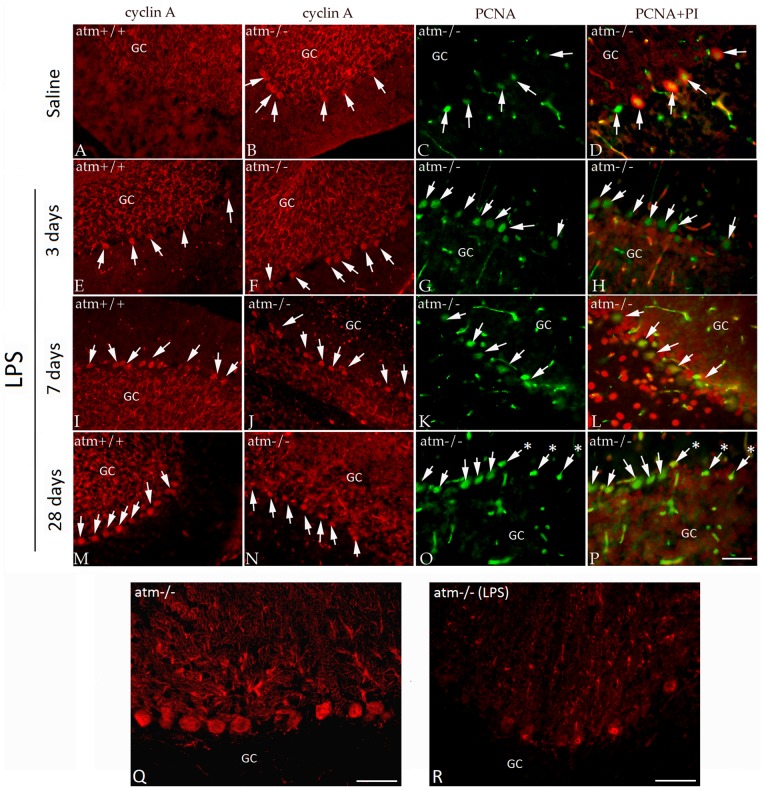
LPS-induced inflammation enhances cell cycle re-entry in *Atm^−/−^* mice. The representative pictures from each group (n = 3–4, repeated 3 times) were shown. In untreated animals (A–D), *Atm^−/−^* mice (B–D) had the expected level of cell cycle protein expression as reported previously; no expression was found in wild type (A). Increased expression of cyclin A (E–F, I–J, M–N, in red) and PCNA (G–H, K–L, O–P, in green) in Purkinje cells were observed after various treatment times. Three days after LPS treatment (E–H), 7 days (I–L) and 28 days (M–P), cell cycle protein expression increased in both genotypes. After prolonged LPS treatment, the cyclin A and PCNA stained *Atm^−/−^* Purkinje cells (N–P) had condensed nuclei suggesting that they were dying (asterisks in O–P). No signs of death were found in the cell cycle positive wild type Purkinje cells (M). To better reveal the total cells, propidium iodide (PI) was used as a counterstain in the PCNA samples (D, H, L, P). In all panels, white arrows indicate cell cycle positive Purkinje cells. Calbindin immunostaining revealed signs of degenerative changes in *Atm^−/−^* Purkinje cells (R), compared to untreated mutants (Q) after LPS challenge. GC = granule cell layer, PI (red in D, H, L, P) was used as a nuclear counterstain. Scale bar = 50 µm.

The expression of cell cycle proteins was significantly increased in the Purkinje cells of all LPS-treated *Atm^−/−^* mice at 3 days (Figure 2F–H), at 7 days (Figure 2J–L) and at 4 weeks (Figure 2N–P) when compared with non-LPS treated *Atm^−/−^* animals (Figure 2B–D). Significantly, in wild type (*Atm* ^+/+^) mice exposed to the same regimen of acute and chronic LPS treatment, the expression of cell cycle proteins also appeared in the nuclei of Purkinje cells (Figure 2E, I, M). In the LPS-treated wild type animals, we found that the number of ‘cycling’ Purkinje cells was greater in the long-term (Figure 2M) than in the short-term (Figure 2E) situation. As expected, no cell cycle protein expression was found in the Purkinje cells of untreated wild type animals (Figure 2A). After 4 days of LPS-induced inflammation, *Atm^−/−^* (Figure 2Q–R) but not wild type (not shown) Purkinje cells began to show a clear loss of morphological integrity assessed by the appearance of their calbindin immunocytochemistry. The nuclei of *Atm* ^−/−^ Purkinje cells exposed to 28 days of LPS treatment were more condensed (arrows with asterisks, Figure 2O–P). Their dendrites also appeared abnormal (Figure 2 Q–R) and gave every indication, based on their calbindin content, that they were slowly atrophying.

Both exposure to low oxygen and chronic LPS administration had qualitatively similar effects on the cerebellar Purkinje cell population – a marked increase in both the number of Purkinje cells expressing cell cycle proteins as well as in the intensity of expression of the immunostaining. Counts of the percentages of cell cycle positive Purkinje cells revealed that in either hypoxia or LPS treated *Atm^−/−^* animals, nearly 26% of the cerebellar Purkinje cells were positive for cell cycle markers such as PCNA or cyclin A. These values should be compared to those reported previously for untreated *Atm^−/−^* animals [12] and reproduced here (10% PCNA^+^; 9% cyclin A^+^). This involvement of more than twice the number of Purkinje cells showing evidence of a neuronal cell cycle is compelling evidence for the impact of a second stressor on the cell cycle status of the vulnerable neurons. Further, the similarity of the LPS and low-oxygen responses suggests that the effects we observed on cell cycle proteins were not specific to any one treatment. We have therefore focused our subsequent efforts solely on the LPS injection paradigm.

**Figure 3:**
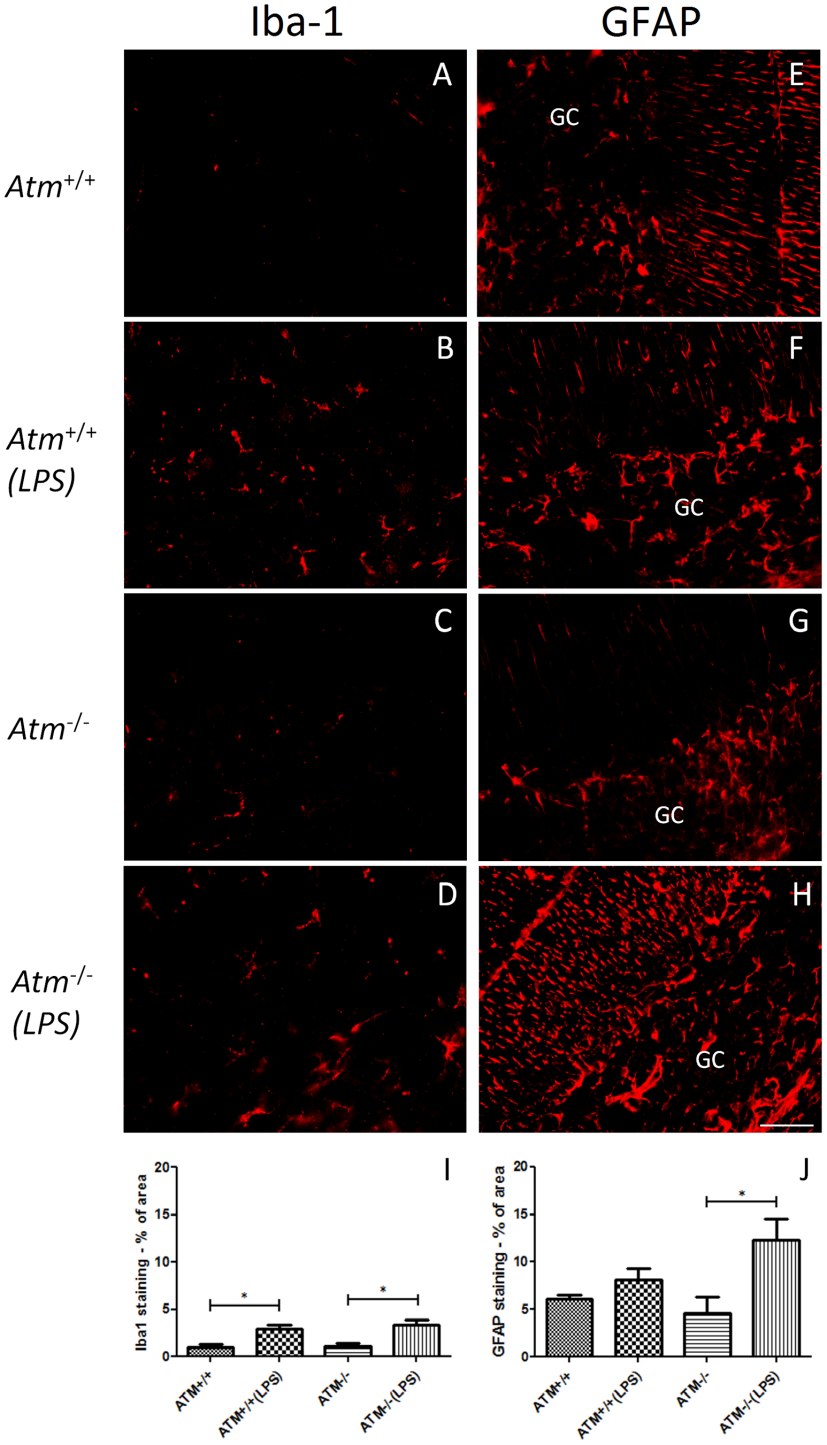
An inflammatory response was observed in both genotypes of LPS-induced mice. The representative pictures from each group (n = 3) were shown. The microglial marker, Iba-1 was used to reveal the morphology of the brain microglia in both wild type (A–B) and *Atm* ^−/−^ (C–D) cerebellum. In saline-treated animals (A, C), the microglia appeared in a typical resting phenotype with thin, spindly processes. After LPS treatment (B, D), the microglia in both genotypes appeared reactive with thicker processes. GFAP, a marker for activated astrocyte, increased only in *Atm* ^−/−^ mice (H) but not in *Atm* ^+/+^ mice (F) under LPS treatment. The quantification of occupied area by Iba-1 (I) and GFAP staining (J). Scale bar = 50 µm.

To monitor the inflammation induced by systemic LPS injection, we immunostained our material with the microglial marker, Iba-1. As we expected, we found evidence of a robust inflammatory response in both wild type and *Atm^−/−^* animals using the reactive morphology of the microglia seen after Iba1 staining (Figure 3). While the Iba-1-positive cells in the untreated brains had thin and spindly processes (Figure 3A, C), the LPS treated brains showed widespread evidence of shortening and thickening of the processes as would be expected during an inflammatory response (Figure 3B, D).

We quantified the non-neuronal responses during the days immediately following the stimulus by repeating the paradigm of daily LPS injections for 4 days followed by sacrifice 24 hours after the last injection. LPS led to a statistically significant increase in Iba1 staining in wild type and *Atm^−/−^* cerebellum (Figure 3I). Cerebellar astrocytes were also affected, as measured by the GFAP coverage of the same regions (Figure 3E–H). In this case, the wild type animals showed only a trend towards increased glial involvement while the increase in GFAP in the mutants was significant (Figure 3J).

### Acute LPS Administration Induces ATM Gene Expression

**Figure 4:**
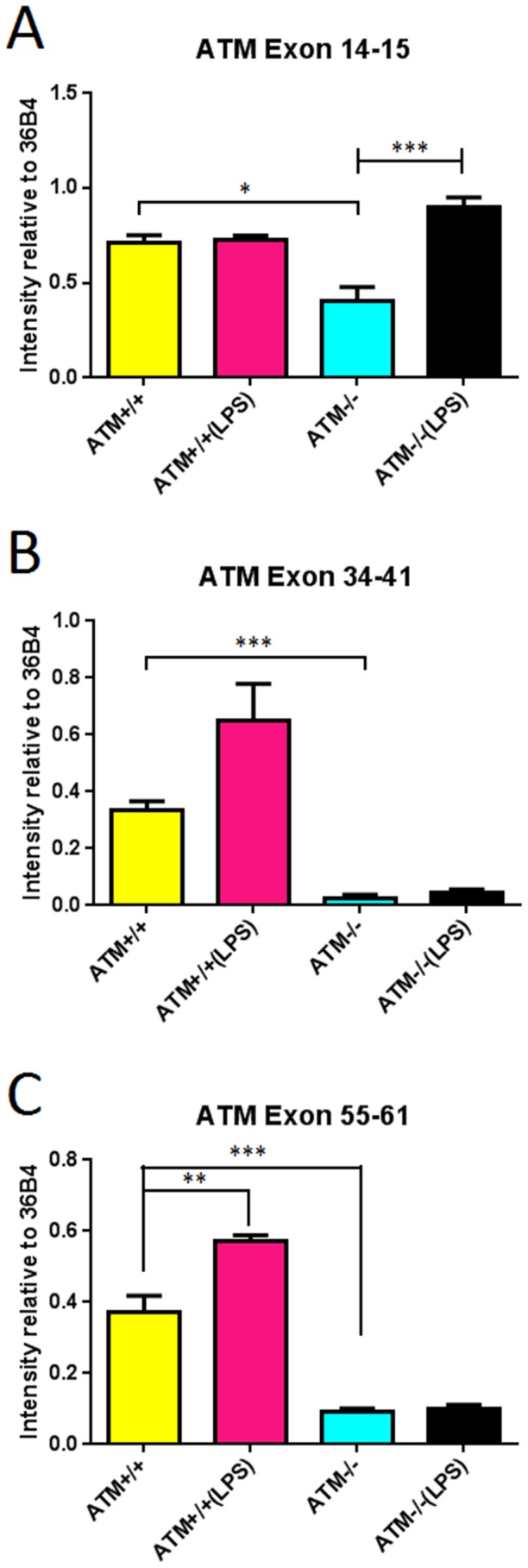
Acute LPS administration stimulates *Atm* expression in both genotypes. *Atm* mRNA was generally reduced in *Atm* ^−/−^ mutants compared with wild type. Three regions of the 10 kb message were analyzed (A) exons 14–15 (B) exons 34–41 and (C) exons 55–61. Experiments were done in 3 animals each group.

We next determined if the transcription of the *Atm* gene itself were sensitive to the challenges imposed by the acute inflammatory challenge of 4 LPS injections. Although the *Atm^tm1Awb^* allele is a true null in most tissues, we have previously shown that a stable population of (truncated) mRNA can be found in mutant brain tissue [13] The *Atm* message is 10kb in length; therefore we assayed RT-PCR fragments from 3 different portions along its total length – exons 14–15, 34–41 and 55–61. Generally, the expression of all three regions of the *Atm* message was significantly lower in *Atm^−/−^* cerebellum than in wild type (Figure 4A–C) although the length of the amplified 34–41 fragment where the mutation was engineered was shorter in *Atm^tm1Awb^* animals compared to wild type [13] After LPS injections the *Atm* message levels trended higher in both genotypes, reaching significance for the wild type only in exons 55–61 and for the mutant only in exons 14–15. Within the limits of our assay, therefore, it appears that one consequence of the inflammatory response is to induce an increase in the expression of the *Atm* gene.

### Cell Death Markers Appear in Purkinje Cells after Inflammatory Treatment

**Figure 5:**
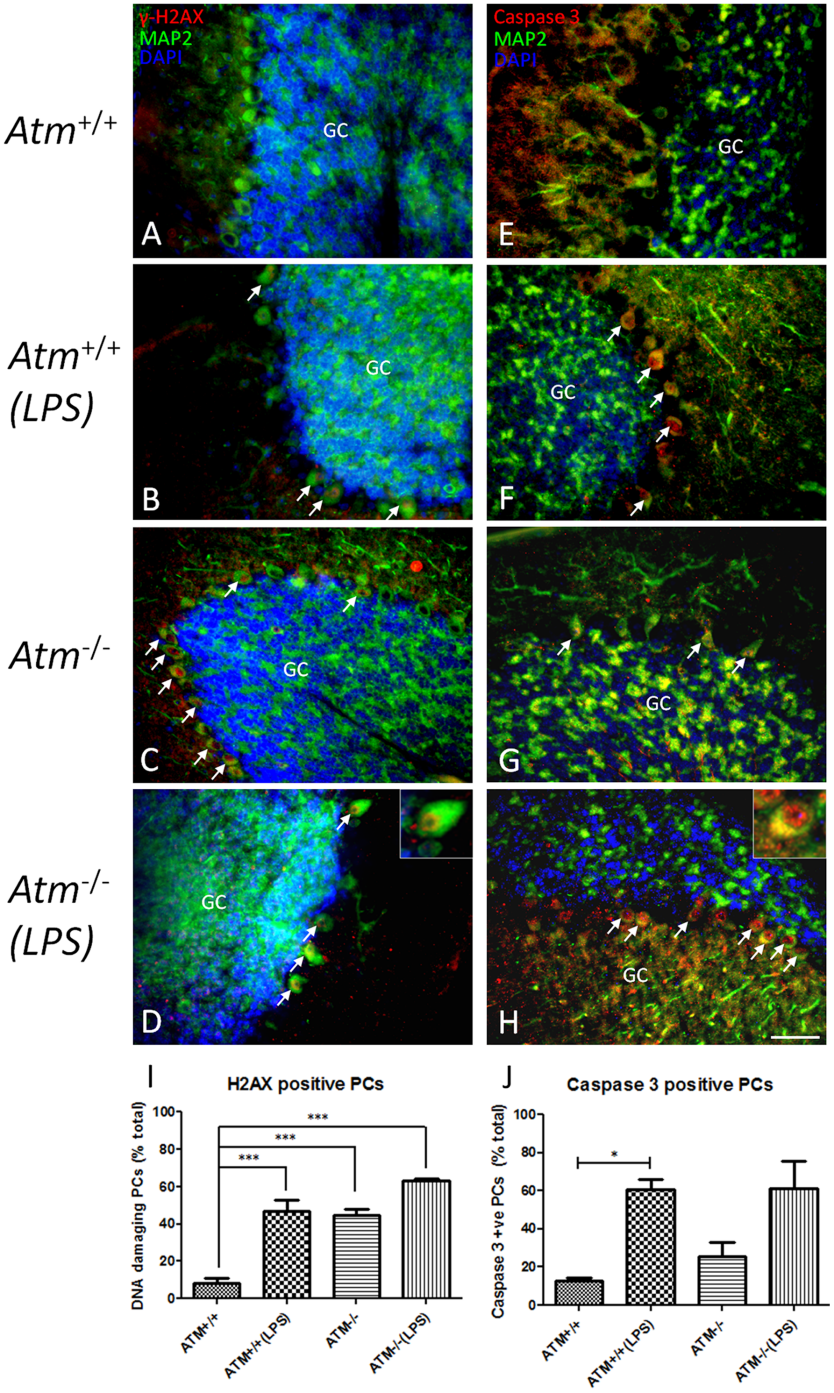
DNA-damage and apoptotic cell markers in *Atm^−/−^* and wild type mice after LPS treatment. The representative pictures from each group (n = 3) were shown. Nuclear localizations of **γ**-H2AX (red, A–D) and cleaved caspase 3 (red, E–H) were identified in saline-treated *Atm^−/−^* mice (C, G) but not in *Atm^+/+^* mice (A, E). Map2 staining (green) was used to identify Purkinje cells. Cell nuclei were revealed with a DAPI (blue) counterstain. Quantification of marker-positive Purkinje cells for **γ**-H2AX (I) and cleaved caspase 3 (J) was performed. In panels A–H, white arrows indicate the Purkinje cells that stained with respective protein markers. Scale bar = 50 µm.

**Figure 6:**
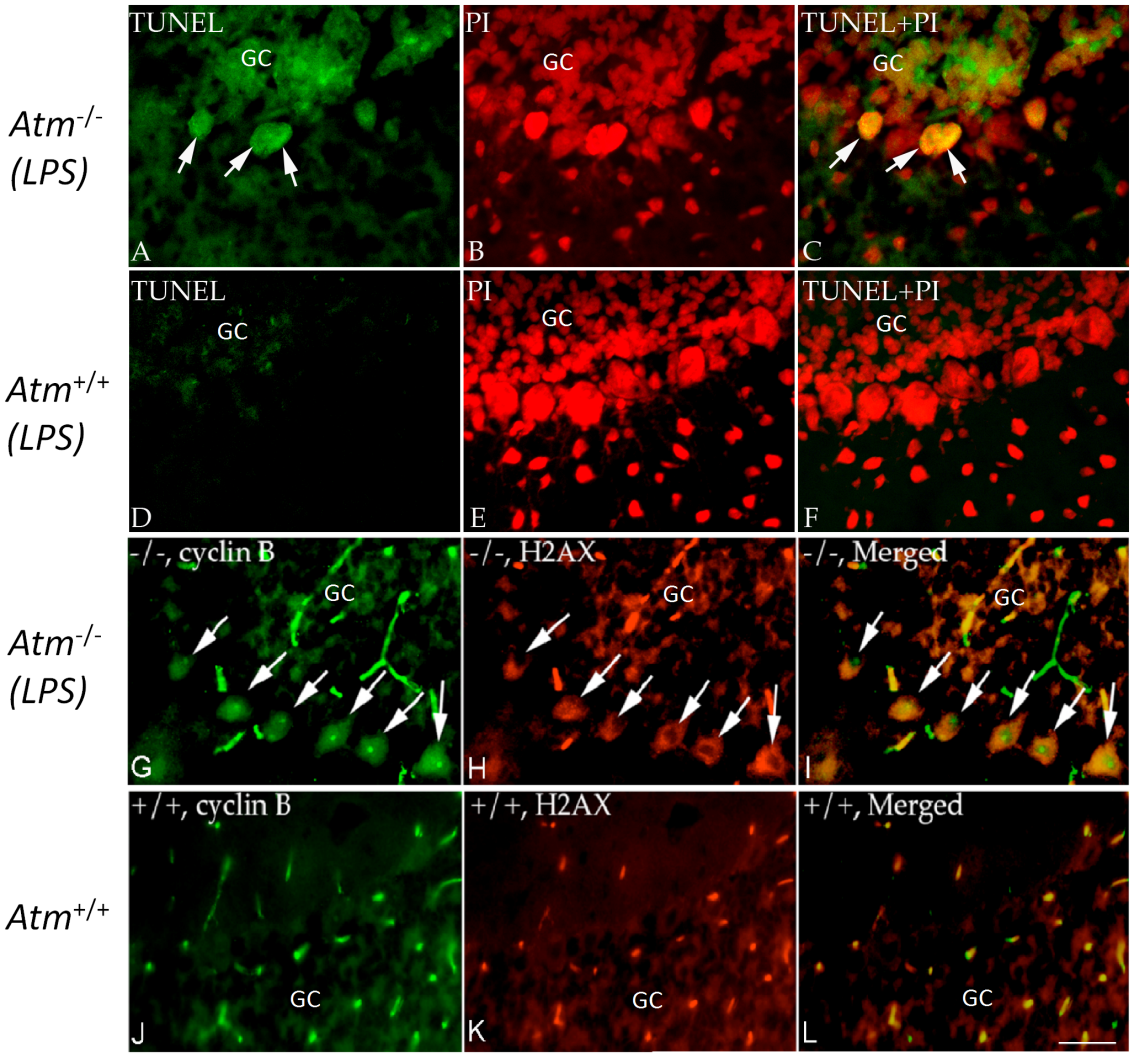
Appearance of cell death markers in *Atm^−/−^* mice after LPS treatment. The representative pictures from each group (n = 3–4, repeated 3 times) were shown. (A–F) TUNEL staining was found in a small number of *Atm^−/−^* Purkinje cells (A–C), but not in wild type controls (D–F) after 3 days of LPS treatment. TUNEL preparations were counterstained with PI. After 4 weeks of LPS treatment, many *Atm^−/−^* Purkinje cells expressed cyclin B (green, G–I) and those that did also co-localized with **γ**-H2AX in mice (red). In wild type controls (J–L) nuclear cyclin B and **γ**-H2AX staining were rare. Scale bar = 50 µm.

We have shown that abortive cell cycle events are associated with neuronal degeneration in human A–T postmortem material [12]. Curiously, despite the presence of cell cycle events, little evidence of neuronal atrophy or loss appears in the Purkinje cell population of the *Atm^−/−^* mouse. Nonetheless, we hypothesized that the doubly challenged neurons might have begun a degenerative program. We stained tissue for evidence of DNA damage and fragmentation that accompanies the apoptotic process using immunostaining for the phosphorylated histone, **γ**-H2AX, cleaved caspase-3 and TUNEL. In both *Atm^+/+^* and *Atm^−/−^* animals treated with LPS for 4 days, we found many examples of Purkinje cells immunopositive for **γ**-H2AX in their nuclei (Figure 5A–D). Immunostaining for cleaved caspase-3, an independent cell death indicator, produced similar results (Figure 5E–H) and quantification confirmed that LPS injection led to a significant increase in both cell death markers (Figure 5I, J). The damage was most robust in *Atm^−/−^* animals injected with LPS. While both of these markers are commonly used as indices of cell death, in animals subjected to chronic LPS treatment (4 weeks) **γ**-H2AX immunopositive Purkinje cells remained present even though there was little indication of a reduction in Purkinje cell density in cerebellar cortex (not shown). TUNEL-positive Purkinje cells were also visible in the LPS injected mutant animals (Figure 6A–C) but not in wild type (Figure 6D–F). Wild type animals injected with saline showed no evidence of DNA breaks by TUNEL (not shown).

To document that the cell cycle-positive cells were the same cells that were engaged in cell death, we double immunostained for cell cycle proteins and cell death markers. Using either cyclin A (not shown) or cyclin B (Figure 6G–I) as markers, Purkinje cells of *Atm^−/−^* animals treated with LPS for 3 days that were cell cycle positive were also positive for **γ**-H2AX. The effect required both the mutant genotype and the LPS injections. There were a few **γ**-H2AX Purkinje cells in untreated *Atm^−/−^* animals (Figure 5). When they occurred, they were always positive for cell cycle markers. Untreated *Atm* ^+/+^ Purkinje cells showed virtually no evidence of either cell cycle or cell death markers (Figure 5A, E, I, J). In LPS-treated animals about 15% of the Purkinje cells are either cyclin A/**γ**-H2AX or cyclin B/**γ**-H2AX double-positive. We found occasional examples of Purkinje cells that were positive for only one of the two markers. These were always immunopositive for cell cycle proteins but not for cell death markers. Significantly, the reverse was never true. No cell that was positive for staining with **γ**-H2AX, TUNEL or activated caspase-3 was found to be negative for cell cycle protein immunostaining.

**Figure 7:**
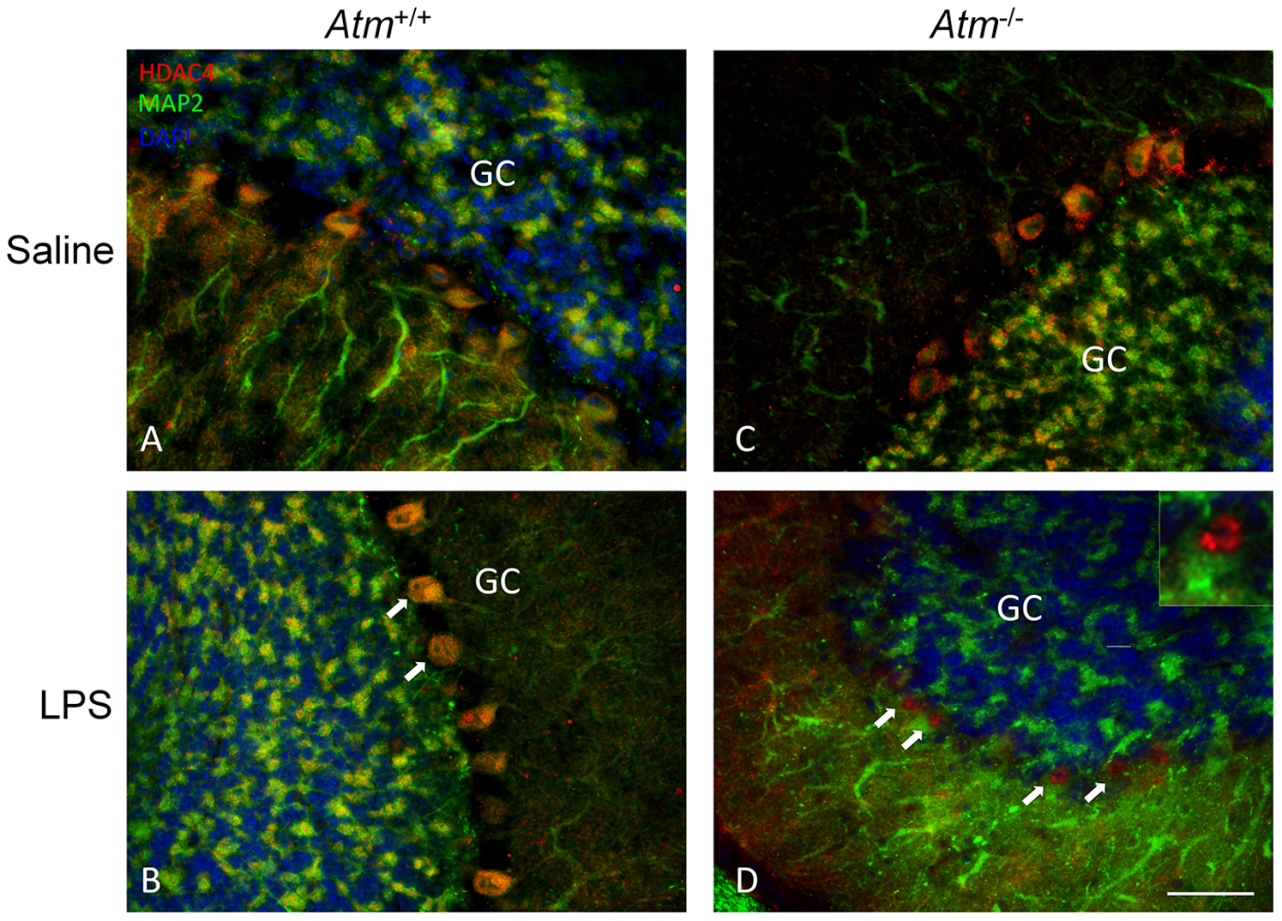
LPS-induced inflammation stimulated nuclear accumulation of HDAC4 in both wild type and *Atm^−/−^* mice. The representative pictures from each group (n = 3) were shown. No expression of nuclear HDAC4 was observed in *Atm^+/+^* mice (A) but was found in *Atm^−/−^* mice (C). After LPS treatment, HDAC4 expression translocated into the nucleus of *Atm^−/−^* Purkinje cells (D) but wild type Purkinje cells (B). White arrows indicate the examples of nuclear HDAC4 in Purkinje cells. One example (white arrow head) is enlarged to show the red fluorescence in the nucleus (D, inset). Scale bar = 50 µm.

We have recently found that in ATM deficiency, the histone deacetylase, HDAC4 is found in Purkinje cell nuclei in addition to its normal cytoplasmic location. Indeed, this translocation is proposed to be an important part of the mechanism of neurodegeneration in the ATM deficient mouse model [6]. We have also shown that, in Alzheimer’s disease, the fraction of hippocampal pyramidal neurons with nuclear HDAC4 increases significantly, suggesting a failure of ATM signaling [11]. To determine the consequences in the cerebellum of an LPS challenge, we immunostained sections from the series of treated animals for HDAC4. As we expected, we found that nuclear localization was stimulated following LPS injection in *Atm^−/−^* (Figure 7C, D) but not in *Atm^+/+^* animals (Figure 7A, B), further suggesting the disproportionate disruption of neuronal homeostasis in *Atm^−/−^* Purkinje neurons under immune challenge.

## Discussion

The results presented here offer a new perspective on the interaction of secondary, environmental stressors with the process of cell cycle events and the degeneration of neurons in ataxia-telangiectasia. The vulnerable neuronal populations in human A–T, those that suffer numerical loss during the disease, are marked by re-expression of cell cycle proteins in many of the surviving neurons before their death [12]. In the ATM-deficient mouse, previous studies from other groups suggest that there is little or no evident loss of any neuronal cell type for the life of the animal [23], though some have questioned this conclusion [24], [25]. If the cell cycle events are taken as evidence for heightened risk of cell death, however, then the mouse perfectly captures the phenotype of neuronal distress of A–T in the human, even in the absence of overt cell loss. Unscheduled cell cycle events are initiated in the homologous populations in both human and mouse (cerebellar Purkinje and granule cells as well as striatal neurons). Given that the other non-neuronal phenotypes of the disease are virtually identical, the question raised by the partial phenotype observed in brain is why the mouse neurons do not die.

Others and our lab have speculated that a cycle-related neuronal death of a mature neuron requires a ‘second hit’ after the cell cycle has been initiated [15], [26]. This concept is borrowed directly from the cancer literature where it has long been proposed that multiple mutations are needed to trigger an oncogenic transformation [27]. In this analogy, just as a prospective tumor cell is protected such that a single mutation is not sufficient to trigger a cancer, the nerve cell appears to have redundant failsafe mechanisms that are called into play to ensure its survival during times of stress. Redundancy such as this has also been proposed as an explanation for the synergistic effects of APP and p73 mutations [28]. The genetic loss of ATM function is apparently sufficient to enable the initiation of a cell cycle process in susceptible Purkinje and striatal neurons, but this event alone is not sufficient to drive a cell to enter the death program. To identify those factors that could serve as the hypothesized second hit, we used inflammation and hypoxia to challenge the compromised *Atm^−/−^* neurons. Our findings demonstrate that vulnerable cells are quite sensitive to these additional challenges.

Inflammation or oxidative stress interacts with the genetic vulnerability caused by ATM deficiency to markedly increase the involvement of cell cycle and cell death processes. These results validate the two-hit model but also underscore its complexity. The complexity arises because the responses of the affected neurons are not consistent with a simple two-step event; cell cycling can be enhanced, without directly inducing cell death. It may be that we did not allow enough time after the hypoxia for the cell death markers to fully develop, but any temporal delay must be at least several hours long.

This identifies a second complexity of the findings. It would appear that death is at best a slow response to cell cycle induction. Unlike the hypoxia experiments, an apoptotic-like process did begin in the inflammatory model. Yet, it was only observed in a fraction of the *Atm^−/−^* Purkinje cells; a few apoptotic markers could also be found in wild type Purkinje cells subjected to the same stressors. We identified cells in the process of death in three ways: the presence of caspase-3, the nuclear presence of phosphorylated histone H2AX (**γ**-H2AX) and the appearance of DNA breakage as measured by TUNEL. Each of these is a frequently used measure of cell death and, in the aggregate, there seems little doubt that the Purkinje cells shown in Figures 5 and 6 would meet criteria for dying neurons. Yet, a comparison of the 4-week and 4-day exposures reveals little decrease in Purkinje cell density. This leads to the suggestion that a death process may have begun, but it is slow to advance to completion. The wild type results even suggest, as have others, that all three markers may be reversible. The dendritic atrophy that we observe in our calbindin immunostained preparations (Figure 2) supports the idea of a degenerative process that is protracted and possibly incomplete. It is noteworthy that we observed no hint of ataxia in any treated animal, another measure of the incomplete nature of the process.

The finding that chronic inflammation can exacerbate the course of A–T is consistent with findings in other neurodegenerative diseases. We used systemic LPS injections in our study, as this is a well-tested model of the inflammatory process. LPS is a cell wall component of gram-negative bacteria, which is known to induce a profound inflammatory response both in the periphery and in the brain [29]. We have every reason to believe that the neurons of the treated mice were exposed to elevated level of inflammatory cytokines. This is based in part on findings from earlier studies, and in part on the observed evidence of microglial and astrocytic reaction (Figure 3). Peripheral administration of LPS can lead to functional impairments that affect both memory and learning [18], [30], [31], as well as to structural damage to elements such as myelin, axons, and the neuronal cell body within the central nervous system [32]. Combrinck et al [33] showed that a systemic challenge with LPS provokes exaggerated IL-1ß synthesis in the CNS with overt signs of sickness and diminished behavioral responses; this also appears to be true in transgenic models of AD [34]–[36]. The response to experimental brain injury such as stroke is exacerbated by the microinjection of the pro-inflammatory cytokine, interleukin-1ß [37], [38]. It is also known that peripheral infection can initiate the synthesis of cytokines within the CNS [39], [40] and an amplified cytokine and inflammatory response may lead to increased neuronal loss and functional deficits [41].

The question of whether brain inflammation is involved in the origins of human A–T is not addressed by our study. Our injections were given to animals 2 months old or older. For reference, puberty in the mouse occurs at 6–8 weeks. Our results are, therefore, not likely to have relevance for the onset of human A–T symptoms at 2–3 years of age (roughly equivalent to 2–3 weeks in mouse). There is no reason to assume that inflammation is a necessary step in the human disease, but it is possible that environmental factors such as inflammation may alter the severity or timing of the onset of symptoms. This possibility is suggested by the observation in *Atm* ^−/−^ mice that LPS injection increases the number and intensity of cell cycle staining and leads to the expression of common markers of cell death.

## Materials and Methods

### *Atm*-deficient Mice

A breeding colony of mice with a targeted disruption of the *Atm^tm1Awb^* gene [42] was obtained from The Jackson Laboratory (Bar Harbor, ME). Generation of mutants was achieved through the mating of heterozygous *Atm^+/−^* males and *Atm^+/−^* females. The mice were maintained on a 129/SvJ genetic background. Genotyping was performed on extracted tail DNA using PCR techniques were described previously [42]. All animals were housed either at Animal Resource Center of Case Western Reserve University Medical School, which is a facility fully accredited by the Association for Assessment and Accreditation of Laboratory Animal Care, or at the accredited Animal and Plant Care Facility of Hong Kong University of Science and Technology. All procedures involving animals were approved by the respective Institutional Animal Care and Use Committees and follow Public Health Service regulations and guidelines (Cleveland) and the Hong Kong Department of Health guidelines (Hong Kong).

### Treatments with Lipopolysaccharide LPS and/or 8% Oxygen

LPS administration: LPS (Escherichia coli serotype 055:B5) was purchased from Sigma-Aldrich (L2880, St. Louis, MO, USA) and dissolved in filtered saline as a stock at concentration of 3 mg/ml. LPS was administrated intraperitoneally to adult mice (2 to 3 month old) of either *Atm* ^+/+^ or *Atm^−/−^* genotype. Three treatment groups were as follows: (1) The first group of animals (3 *Atm* ^+/+^ and 3 *Atm^−/−^*) received daily intraperitoneal (i.p.) injections of LPS (1 mg/kg) for 4 days to elicit an acute systemic inflammatory response. A control group (3 *Atm* ^+/+^ and 3 *Atm^−/−^*) were treated on the same schedule, but injected with filtered saline only. During the treatment, mice were monitored carefully. Mice were killed 24 h after the last injection; the brains were dissected and the tissues prepared described below. (2) A second experiment involved groups of 3 animals; each was given a single i.p. injection of LPS, but sacrifice and subsequent analysis did not occur until one week later. This experiment was repeated 3 times. (3) A third experiment involved groups of 3 animals; each was given weekly intraperitoneal injection of LPS once a week for 4 weeks and animals were sacrificed within 24 h after the final injection. This experiment was also repeated 3 times.

### Tissue Preparation and Histology

Animals were deeply anesthetized with Avertin (0.02 cc/g body weight) and transcardially perfused with 1.5 ml cold PBS, followed by approximately 30 ml of 4% paraformaldehyde in 0.1M phosphate buffer saline (PBS). After perfusion, the brain was dissected out and was transferred to 30% sucrose solution at 4°C overnight for cryoprotection. After bisecting along the midline, the brain was embedded in OCT; 10 µm cryostat sections were cut and allowed to air dry on pre-coated *SuperPlus* glass slides.

### Single or Double Immunocytochemistry

#### Primary antibodies

The proliferating cell nuclear antigen (PCNA) mouse monoclonal antibody (#2586; Cell Signaling Technology, USA) was diluted 1∶2000 in 10% goat serum/PBS blocking buffer before use. The rabbit polyclonal cyclin A antibody (sc-751; dilution 1∶100, Santa Cruz Biotechnology, USA) was raised against amino acids 1–432 representing full length cyclin A of human origin. Polyclonal rabbit **γ**-H2AX antibody against the residues 100–200 of human **γ**-H2AX (phosphorylated at S139) was purchased from Abcam (Cambridge, UK, ab2893; dilution, 1∶250). Polyclonal rabbit antibody against cleaved caspase-3 (#9664; Cell Signaling Technology, USA) and polyclonal rabbit antibody against HDAC4 (ab1437; Abcam, Cambridge, UK) were used at dilutions of 1∶400 and 1∶200, respectively. To detect whether inflammation had been induced efficiently in the CNS after LPS injection, adjacent sections were stained with Iba1 antibody to detect activated microglial cells (ab7260; working dilution, 1∶2000, Abcam, Cambridge, UK) and GFAP antibody to reveal activated astrocytes. Terminal deoxynucleotidyltransferase (TdT)-mediated dUTP nick end labeling (TUNEL) was performed with a kit purchased from Millipore (S7111; Billerica, USA) according to the manufacturer’s instructions. Some sections were co-stained with polyclonal chicken antibody against MAP2 (ab5392; dilution 1∶5000, Abcam, Cambridge, UK) to visualize the neurons for cell counting.

#### Secondary antibodies

Flurochrome-conjugated secondary antibodies (Alexa 488 or 594) were raised against either rabbit or mouse immunoglobulin (1∶500, Invitrogen, Grand Island, NY) or chicken immunoglobulin (1∶500, Jackson Laboratories, West Grove, USA).

#### Immunostaining protocol

Single or double fluorescence immunocytochemistry was performed on mouse brain cryosections according to standard methods. Sections were rinsed in PBS, followed by pretreatment at 95–100°C in 0.1M citrate buffer for 8–10 minutes. After the slides had cooled in buffer for 45–60 minutes at room temperature, slides were rinsed in PBS. Sections were incubated in 10% goat serum in PBS for 1 hour at room temperature to block non-specific binding. All primary antibodies were diluted in PBS containing 0.3% Triton X-100 and 10% goat serum and then were applied to sections and incubated overnight at 4°C. After rinsing in PBS, they were incubated for 2 hours at room temperature with secondary antibody conjugated with fluorescent Alexa dyes. Sections were then rinsed in PBS and counter-stained with DAPI for 5 minutes at room temperature. After rinsing, all sections were mounted with anti-fading fluorescence media (Jackson Laboratories) under a glass coverslip. All experiments were conducted in triplicate.

#### Reverse transcription

Whole cerebellum tissues were homogenized in lysis buffer and processed to RNA extraction using PureLink RNA Mini Kit (Invitrogen) according to manufacturer’s protocol. Subsequently, 500 ng of total RNA was reverse transcribed into total cDNA using ThermoScript RT-PCR System (Invitrogen). The mRNA level of 36B4 was used as an internal control. The following sets of primers were used:

ATM exons 14–15

5′-gattacaagttctgaaaccc-3′ (sense)

5′-gcttggtatgtatacacaagc-3′(antisense)

ATM exons 34–41

5′-ggaaggtgtttgggagaaatagg-3′ (sense)

5′-ccgtattctagtaagggatgtaacattttc-3′ (antisense)

ATM exons 55–61

5′-gatgtaagatggtgaaggacatgg-3′ (sense)

5′-cacagcaccttctgaagaccaattc-3′ (antisense)

The PCR program was set to 55°C for 30 min and 94°C for 3 min, followed by 35 cycles of 94°C for 30 s, 55°C for 30 s, and 68°C for 1–2 min with an extension at 68°C for 10 min. The PCR products were analyzed on 1.5% agarose gels stained with ethidium bromide and visualized under ultraviolet light.

### Cell Counts

Five fields were randomly chosen for quantification using a 20× objective on an Olympus or a Leica fluorescent microscope. In each field the total number of Purkinje cells was determined using either MAP2 or calbindin staining. We then separately determined the numbers of Purkinje cells that were positive for these experimental markers: PCNA, cyclin A, **γ**-H2AX, cleaved caspase-3 or HDAC4. The percentage of the positive cells with cell cycle markers or cell death markers were counted out of total Purkinje cells. Percentages of area occupied by Iba-1 or GFAP positive glial cells were calculated using software ImageJ (National Institutes of Health). One-way ANOVA (Prism, GraphPad software, Version 5) was used to determine the differences in staining patterns between different groups. *p*<0.05 was considered statistically significant.
