# Correction: The Interaction of the *Atm* Genotype with Inflammation and Oxidative Stress

**DOI:** 10.1371/journal.pone.0130645

**Published:** 2015-06-17

**Authors:** Yan Yang, Chin Wai Hui, Jiali Li, Karl Herrup

The following information is missing from the Funding section: This study was supported by the Research Grants Council (RGC) of Hong Kong (GRF660813).
